# Phylogeny and expression pattern analysis of TCP transcription factors in cassava seedlings exposed to cold and/or drought stress

**DOI:** 10.1038/s41598-017-09398-5

**Published:** 2017-08-30

**Authors:** Ning Lei, Xiang Yu, Shuxia Li, Changying Zeng, Liangping Zou, Wenbin Liao, Ming Peng

**Affiliations:** 1 Institute of Tropical Bioscience and Biotechnology, Chinese Academy of Tropical Agricultural Sciences, Haikou, 571101 China; 2 Institute of Tropical Agriculture and Forestry, Hainan University, Haikou, 570228 China; 3 National Key Laboratory of Plant Molecular Genetics and National Center for Plant Gene Research (Shanghai), Institute of Plant Physiology and Ecology, Shanghai Institutes for Biological Sciences, Chinese Academy of Sciences, Shanghai, 200032 China; 4 Present Address: Department of Biology, University of Pennsylvania, Philadelphia, PA 19104 USA

## Abstract

The TCP transcription factors usually act as integrators of multiple growth regulatory and environmental stimuli. However, little is known about this gene family in the important tropical crop cassava (*Manihot esculenta*). In this study, 36 *TCP* genes were identified and renamed based on cassava whole-genome sequence and their sequence similarity with *Arabidopsis TCPs*. Typical TCP domains were detected in these proteins by multiple sequence alignment analysis. Evolutionary analysis indicated that *MeTCPs* could be divided into 8 subgroups, which was further supported by gene structure and conserved motif analyses. qRT-PCR analysis revealed tissue-specific and hormone-responsive expression patterns of *MeTCP* genes. Moreover, with global expression and promoter analysis, we found that *MeTCPs* showed similar or distinct expression patterns under cold and/or drought stress, suggesting that they might participate in distinct signaling pathways. Our study provides the first comprehensive analysis of *TCP* gene family in the cassava genome. The data will be useful for uncovering the potential functions of *MeTCP* genes, and their possible roles in mediating hormone and abiotic stress responses in cassava.

## Introduction

As sessile organism, plant growth and yield are strongly influenced by environmental stimuli such as cold and drought^1^. To respond and adapt to these conditions, plants develop various mechanisms at both physiological and biochemical levels^2^. It has been well established that many adaptation processes are regulated by stress-responsive gene expression^1, 3^. Transcription factors (TFs), which are a diverse family of regulatory proteins with DNA-binding domains, play a central role in mediating various aspects of cellular processes by regulating gene expression through interacting with cis-elements in the promoter regions of various downstream genes^4, 5^. Series studies previously have uncovered a group of TF genes, such as *AP*2/*ERF*, *MYB* and *bZIP*, which participate in various stress-induced physiological processes and regulatory networks in higher plants^6, 7^.

*TCP* genes encode plant-specific transcription factors, which are named after the first four functionally identified members: *TB1* (*TEOSINTE BRANCHED 1*) in *Zea mays*, *CYC* (*CYCLOIDEA*) in *Antirrhinum majus*, and *PCF1* and *PCF2* (*PROLIFERATING CELL FACTORS 1* and *2*) in *Oryza sativa* ^8^. Typically, N-terminus of this class of transcription factors exhibits a highly conserved TCP domain, which contains a non-canonical basic-Helix-Loop-Helix (bHLH) structure involved in DNA binding, protein-protein interaction and protein nuclear localization^9, 10^. Based on the homology of the TCP domains, TCP proteins can be further divided into two major subfamilies, Class I (represented by the rice PCF proteins) and class II (represented by CYC and TB1)^8, 11^. The DNA-Binding site selection assays revealed that the consensus binding sequences for these two classes are slightly different, but overlapping with GGNCCC sequences. The DNA binding sequence for class I is GGNCCCAC while class II prefer to bind the DNA motif G(T/C)GGNCCC^10^. Both class I and class II include TCPs that can function as transcriptional activators and repressors^12^.

Increasing evidences have indicated that proteins of TCP family take part in the regulation of many biological processes during plant growth and development, including plant architecture^12, 13^, leaf morphogenesis^14–16^, hormone pathways^13, 17–19^ and response to environmental stimuli among various species^20–22^. For example, studies in *Arabidopsis* suggest that *AtTCP14* appears to function in regulating embryonic growth of seeds^23^. *AtTCP15*, along with its closest homolog *AtTCP14*, regulates cell proliferation in the developing leaf blade and specific floral tissues, and also modulates gibberellins and auxin responses^17, 24^. The expression of a repressor form of *AtTCP11* caused pleiotropic developmental alterations^25^. *AtTCP2*, *AtTCP3*, *AtTCP4*, *AtTCP10* and *AtTCP24* have been earlier identified as targets of miRNA319, and are required for leaf development, petal growth, cell wall synthesis and JA synthesis^14, 26, 27^. Creeping bentgrass (*Agrostis stolonifera*) plants overexpressing Osa-miR319a, in which four putative target genes, *AsPCF5*, *AsPCF6*, *AsPCF8*, and *AsTCP14* were down-regulated, displayed morphological changes and exhibited enhanced drought and salt tolerance associated with increased leaf wax content and water retention^20^. In rice, overexpression of *OsTCP19* led to developmental abnormalities like fewer lateral root formation and contributed to better stress tolerance^21^.

Widely cultivated in tropical area, cassava is considered as one of the most important economic crops worldwide, providing starch for food, feed and energy production^28–30^. Cassava can effectively utilize light, heat and water resource; it is resistant to drought but sensitive to low temperature^31^. Cold and drought stress severely limit cassava plant growth and yield^32^. Thus, uncovering the mechanisms underlying the resistance of cassava to these stresses may provide candidate genes for genetic improvement of stress resistance for cassava. Previously, we performed strand specific RNA sequencing for cassava TMS60444 shoot apices and young leaves under cold and PEG-induced drought stress, providing an excellent resource for analysis of stress responsive genes globally.

To date, numerous *TCP* family members have been identified in various species^33–45^. However, no evidence is available regarding the *TCP* family in cassava. Due to the critical role of *TCP* transcription factors in the control of plant development and abiotic stress responses, we performed for the first time the comprehensive analysis of the *MeTCP* transcription factor family in cassava. In the present study, a total of 36 non-redundant *MeTCP* transcription factor encoding genes were identified in cassava genome and were subsequently subjected to a systematic analysis, including phylogenetic relationships, gene structure, conserved motif and expression pattern among different tissues and hormone treatments. We also analyzed the expression of the *MeTCP* genes under normal growth conditions and various abiotic stressors. On the basis of the expression profiles of *MeTCP* genes and the phylogenetic analysis among the TCP domain proteins in *Arabidopsis*, rice and cassava, the functions of *MeTCPs* were predicted. Taken together, our genome-wide analysis of *MeTCP* gene family will contribute to future studies on the functional characterization of MeTCP proteins in cassava, as well as the identification and comprehensive analysis of the *TCP* transcription factor family in other species.

## Results

### Identification of *TCP* genes in cassava

In order to extensively identify cassava *TCP* genes, two search strategies were used in this study: first, we used the protein sequences of all 24 *TCP* genes from *Arabidopsis* ^33^ as BLASTp queries to perform multiple searches against the latest whole genome of cassava (http://www.phytozome.net/cassava); then, the TCP domain (PF03634)^46^ was employed as query to perform a blast search against the same cassava genome database. After discarding redundant sequences, 36 candidate *TCP* genes were identified in cassava, and further conserved domain detection confirmed that all the identified *TCPs* harbor the conserved TCP domain that is the basic characteristics of this family. Due to the lack of standard annotation designated to the 36 cassava *TCP* genes, we named them *MeTCP2* to *MeTCP23* according to the *Arabidopsis* TCP proteins with highest sequence similarity. The length of the 36 newly identified TCP proteins varied from 73 to 562 amino acid residues and the relative molecular weight ranged from 8.3 to 58.1 kDa, with isoelectric points in the range of 5.53–10.08. The grand average of hydropathy (GRAVY) of this family genes showed that all of the proteins had a negative value, indicating that all the MeTCP proteins were hydrophilic (Table 1).

**Table 1:** *TCP* genes identified in cassava genome.

Gene ID	Name	Length(aa)	MW(Da)	PI	Gravy	Genomic locus
Manes.01G187000.1	MeTCP15a	392	42214.9	9.32	−0.643	Chromosome01:28455200..28457428 forward
Manes.01G263300.1	MeTCP9a	366	38916.33	8.94	−0.283	Chromosome01:33561357..33563642 forward
Manes.01G020700.1	MeTCP18a	387	44030.35	7.93	−0.773	Chromosome01:3502035..3504116 forward
Manes.01G094200.1	MeTCP13a	350	38533.91	8.37	−0.667	Chromosome01:21880606..21882675 forward
Manes.02G055900.1	MeTCP13b	361	39880.76	9.06	−0.63	Chromosome02:4209171..4211393 forward
Manes.02G066400.1	MeTCP12	383	42814.84	9.78	−0.569	Chromosome02:4925458..4927250 reverse
Manes.02G194200.1	MeTCP23a	425	45005.56	7.98	−0.572	Chromosome02:15997522..15999455 forward
Manes.04G016700.1	MeTCP11d	240	26058.47	8.98	−0.394	Chromosome04:1959602..1961886 forward
Manes.04G016800.1	MeTCP11b	155	16551.84	9.45	−0.415	Chromosome04:1966346..1966813 forward
Manes.04G088500.1	MeTCP20a	312	33848.59	7.22	−0.782	Chromosome04:22580287..22581884 reverse
Manes.05G041000.1	MeTCP9b	346	36898.79	7.8	−0.325	Chromosome05:2911279..2913198 forward
Manes.05G100100.1	MeTCP15b	388	41706.51	8.23	−0.582	Chromosome05:8461910..8463076 reverse
Manes.05G123700.1	MeTCP20c	73	8314.64	10.08	−1.034	Chromosome05:14666983..14667397 forward
Manes.05G119300.1	MeTCP18b	372	42181.38	8.12	−0.748	Chromosome05:12207088..12208206 reverse
Manes.06G072800.1	MeTCP18c	416	47024.77	9.48	−0.879	Chromosome06:18738590..18740594 forward
Manes.06G083400.1	MeTCP5a	340	37976.22	8.47	−0.611	Chromosome06:19670814..19672682 reverse
Manes.06G093900.1	MeTCP15c	396	42673.9	7.37	−0.73	Chromosome06:20627278..20631189 forward
Manes.06G141800.1	MeTCP19	358	37747	5.53	−0.478	Chromosome06:24669381..24671688 reverse
Manes.07G022400.1	MeTCP8a	550	56832.22	7.55	−0.646	Chromosome07:2093408..2096799 forward
Manes.08G009200.1	MeTCP11a	185	19802.37	6.97	−0.497	Chromosome08:675290..675847 reverse
Manes.09G051000.1	MeTCP16	403	43569.25	7.58	−0.11	Chromosome09:6817330..6819454 forward
Manes.10G120400.1	MeTCP8b	562	58079.71	8.46	−0.621	Chromosome10:23214679..23218733 reverse
Manes.11G083000.1	MeTCP20b	307	32859.71	8.99	−0.671	Chromosome11:11404469..11406607 forward
Manes.11G108500.1	MeTCP7	273	27679.86	9.72	−0.271	Chromosome11:20090517..20092799 forward
Manes.11G149000.1	MeTCP11c	198	21355.38	9.43	−0.335	Chromosome11:26034972..26035568 reverse
Manes.12G007700.1	MeTCP20d	299	32367.12	9.41	−0.702	Chromosome12:722180..724162 reverse
Manes.13G008300.1	MeTCP20e	282	30554.12	9.52	−0.656	Chromosome13:815757..817107 reverse
Manes.13G138300.1	MeTCP3b	343	37809.47	5.9	−0.782	Chromosome13:26570479..26573043 reverse
Manes.14G058400.1	MeTCP3a	336	36987.57	5.87	−0.699	Chromosome14:4620482..4621572 reverse
Manes.14G077200.1	MeTCP15d	398	42850.16	7.39	−0.716	Chromosome14:6245053..6246249 reverse
Manes.14G086500.1	MeTCP5b	387	43276.03	7.21	−0.713	Chromosome14:6949867..6951866 forward
Manes.14G097000.1	MeTCP18d	474	52697.96	9.31	−0.782	Chromosome14:7827437..7828861 reverse
Manes.15G091000.1	MeTCP4	422	45753.27	6.17	−0.636	Chromosome15:6736704..6738513 reverse
Manes.15G123800.1	MeTCP2a	481	52167.1	7.86	−0.886	Chromosome15:9374446..9382168 reverse
Manes.17G072800.1	MeTCP2b	481	52472.3	7.06	−0.926	Chromosome17:21184852..21198102 reverse
Manes.18G103100.1	MeTCP23b	425	45159.97	6.81	−0.563	Chromosome18:9144633..9147156 forward

### Phylogenetic analysis of the *MeTCP* genes

**Figure 1:**
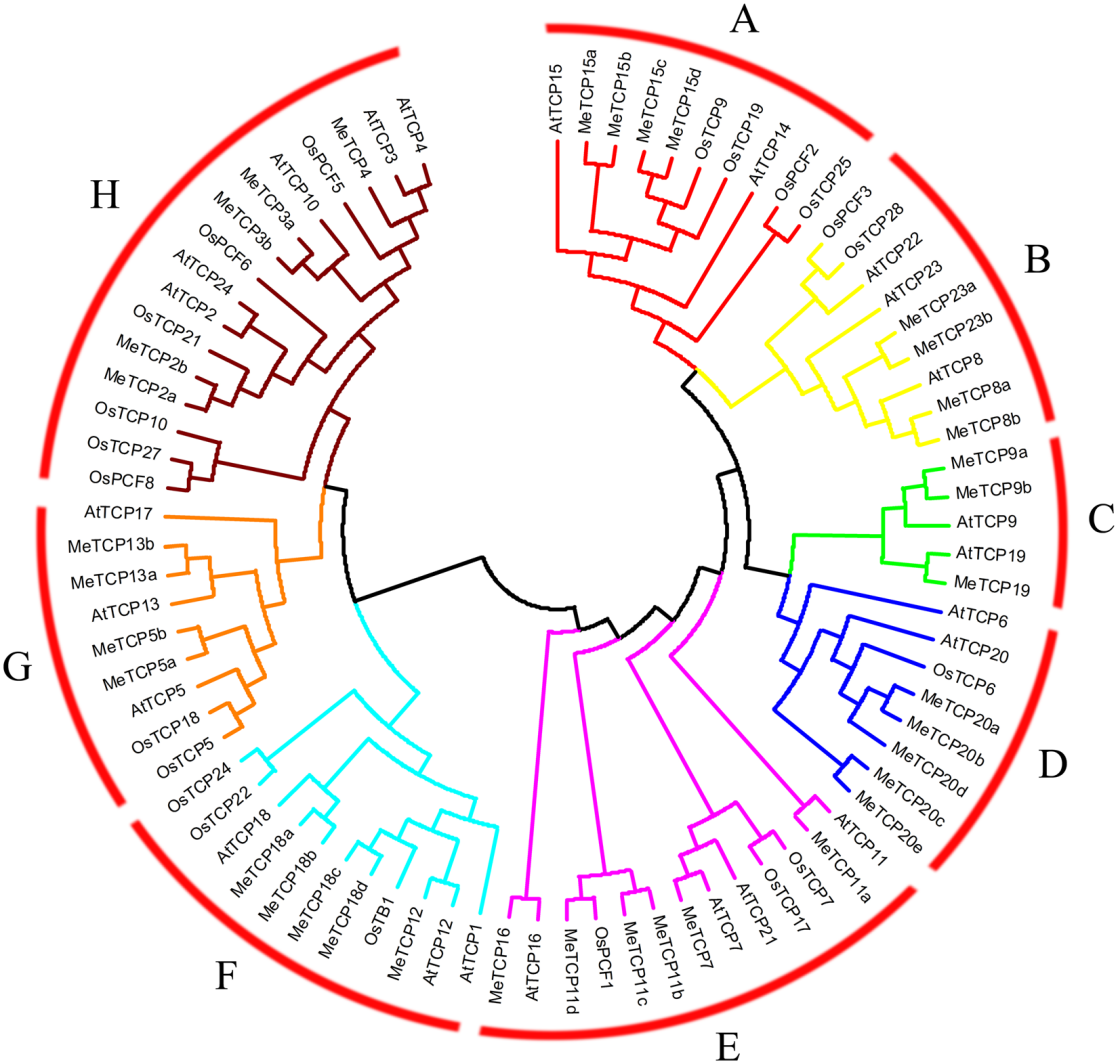
Phylogenetic relationships of TCP transcription factors from cassava, *Arabidopsis* and rice. A total of 36 *MeTCPs* from cassava, 24 *AtTCPs* from *Arabidopsis* and 22 *OsTCPs* from rice were used to construct the Neighbor-Joining tree by MEGA 6.0 with 1000 bootstrap based on the full length sequences of *TCPs*. The eight subgroups are indicated with different colors.

To characterize the evolutionary and phylogenetic relationships between cassava *TCP* genes and other known *TCPs*, an unrooted Neiboring-Joining (NJ) tree was constructed on the basis of multiple sequence alignment of 36 MeTCP complete protein sequences with 24 and 22 TCP protein sequences from *Arabidopsis* and rice, respectively (Fig. 1). At deep nodes, the phylogenetic relationship was unclear and the bootstrap values were low as a result of relatively large number of sequences. To verify the reliability of our phylogenetic tree, we also build the phylogenetic trees of TCP transcription family with Minimal Evolution methods (Fig. S1). The tree topologies were robust within different tree-building methods, except at the deep nodes. Considering these results, the NJ tree was employed for further study.

Based on the bootstrap value of clade and topology of the tree, the MeTCP proteins could be distributed into 8 distinct groups, designated as Group A to Group H. In general, *TCPs* from cassava have closer relationships with the *TCPs* from dicot plant *Arabidopsis* than that from monocot plant rice, which is accord with the current understanding of plant evolutionary history. Additionally, the *TCP* genes showed an interspersed distribution in most clades, which is consistent with those found in previous analyses of *TCP* in *G. raimondii* and *Citrullus lanatus* ^35, 43^, indicating that the TCP family expanded before the divergence of the lineages. However, the *TCP* genes were not evenly distributed in some clades, such as the largest clade Group H has the maximum 7 members, whereas Group C contains only 3 MeTCP genes from cassava, suggesting the existence of a diversified *MeTCP* family in cassava with diverse functions (Fig. 1). Remarkably, many *Arabidopsis TCP* genes had more than three counterparts in cassava, such as *MeTCP11*, *MeTCP15*, *MeTCP18* and *MeTCP20*, indicating that *MeTCP* genes duplicated after the divergence of cassava and *Arabidopsis*. It also suggests that higher number of genes in cassava as compared to *Arabidopsis* is the result of more gene duplication events in cassava or higher frequency of retaining copies after duplication. Group C contained three cassava *TCPs*, two *Arabidopsis* members but there were no *TCP* from rice, implying this group was either acquired after the divergence of monocots and dicots or lost in rice. Remarkably, 5 of the group H members (*AtTCP2*, *AtTCP3*, *AtTCP4*, *AtTCP10*, and *AtTCP24*) are post-transcriptionally targeted by miRNA319 in *Arabidopsis*. The closest homologs of these *Arabidopsis* genes in cassava are these five genes: *MeTCP2a*, *MeTCP2b*, *MeTCP3a*, *MeTCP3b* and *MeTCP4*, all containing putative target site of miR319^47, 48^. This suggests that regulation of leaf development by a redundant set of miRNA-regulated homologous *TCP* genes occurs in cassava.

### Gene structure and conserved motifs of cassava TCPs

**Figure 2:**
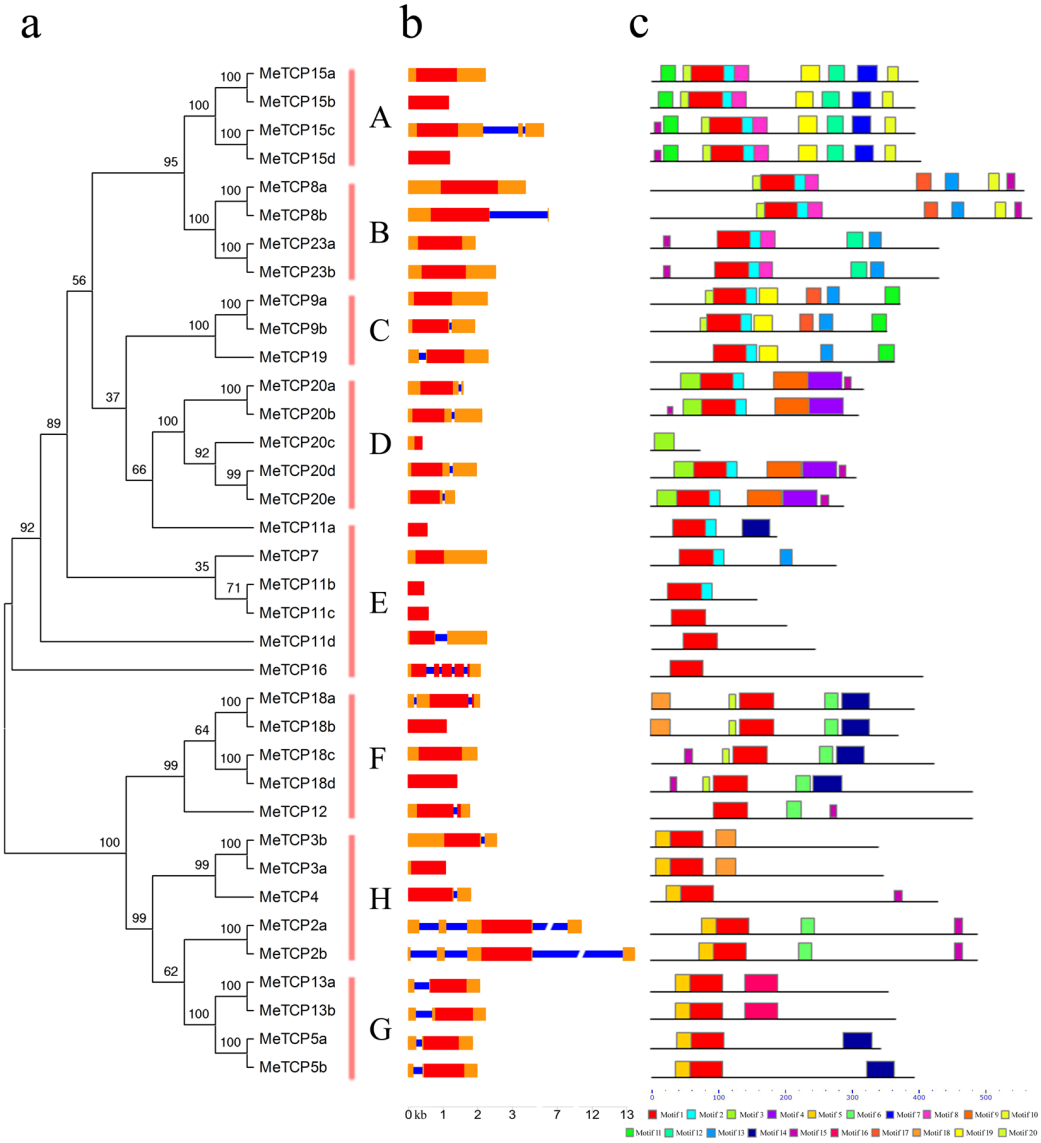
The gene structure and conserved protein motifs of *MeTCP* genes according to the phylogenetic relationship. (**a**) The unrooted phylogenetic tree of all *TCP* genes in cassava was constructed using Neighbor-Joining method and the bootstrap test was performed with 1,000 iterations. (**b**) The gene structure with exon/intron organization of TCP genes of cassava. The orange boxes represent 5′-UTR or 3′-UTR, red boxes represent exons and blue lines indicate introns. (**c**) The conserved protein motifs in the TCP family were identified using MEME program. Each motif is indicated with a specific color.

To further examine the structural features of cassava *TCP* genes, we investigated the exon/intron structures of individual *MeTCP* genes by alignment of cDNA sequences and corresponding genomic DNA sequences. Additionally, we also built an unrooted phylogenetic tree with MeTCP protein sequences (Fig. 2a), to determine whether the gene structure of MeTCPs is consistent with the phylogenetic subfamily. As illustrated in Fig. 2b, the number of introns of *MeTCP* genes varied from 0 to 4. For instance, 32 out of 36 *MeTCP* genes had no intron, while the other *MeTCP* genes possess 1–3 introns, with the exception of *MeTCP16* containing four introns. As expected, most of *MeTCP* genes in the same subfamily showed similar exon-intron distribution patterns in terms of exon length and intron number, which supports their close evolutionary relationship and the classification of subgroup. However, *MeTCP* genes in group H showed great variability in intron number and exon length.

To obtain more insights into the diversity of motif compositions among MeTCPs, conserved motifs were predicted by using MEME program^49^, and annotated by ScanProsite program^50^. A total of 20 conserved motifs in the MeTCP proteins, designated as motif 1 to motif 20, were captured by MEME (Fig. 2c, Fig. S2). The results showed that the only motif that hit for the database was the conserved TCP domain (motif 1). TCP domain was found in all MeTCPs, except MeTCP20c, which contained a truncated TCP domain. In general, MeTCP proteins clustered in same subgroup share similar motif composition, while high divergence was observed among different subgroups. This observation indicated that the MeTCP members within the same subgroup may have redundant functions and that some motifs may contribute to the specific function of that subfamily, which is in agreement with the previous report^35, 41^. According to 36 MeTCP sequence features within the TCP domain, we determined that *MeTCPs* from Group A, B, C, D and E belong to class I subfamily while the other *MeTCPs* belong to class II subfamily, as for all species so far. As reported earlier, the group members belonged to class I subfamily have extended homology C-terminal from the TCP domain, while the class II subfamily has an extended basic region, and all groups have internally conserved, but distinct loop region sequences^25, 51^. The motif analysis also showed that sequence conservation outside the TCP domain was low and sequence length on both sides of the TCP domain varied greatly, resulting in proteins ranging from 73 (MeTCP20c) to 563 (MeTCP8b) amino acids. For example, MeTCP20c, the smallest predicted protein, is probably truncated by a frame shift mutation that cause premature termination, since sequence homology with *Arabidopsis* TCP20 extends well beyond the stop codon. This result is similar to SlTCP27, which encode the smallest TCP protein with 113 amino acids in tomato^36^. Although MeTCP20c lacks the conserved C-terminal part of the TCP domain, which may alter its DNA binding ability, experimental evidences are required to establish the precise role of truncated TCP domain in the regulation of MeTCP20c activity.

### Expression analysis of *MeTCP* genes in different tissues

**Figure 3:**
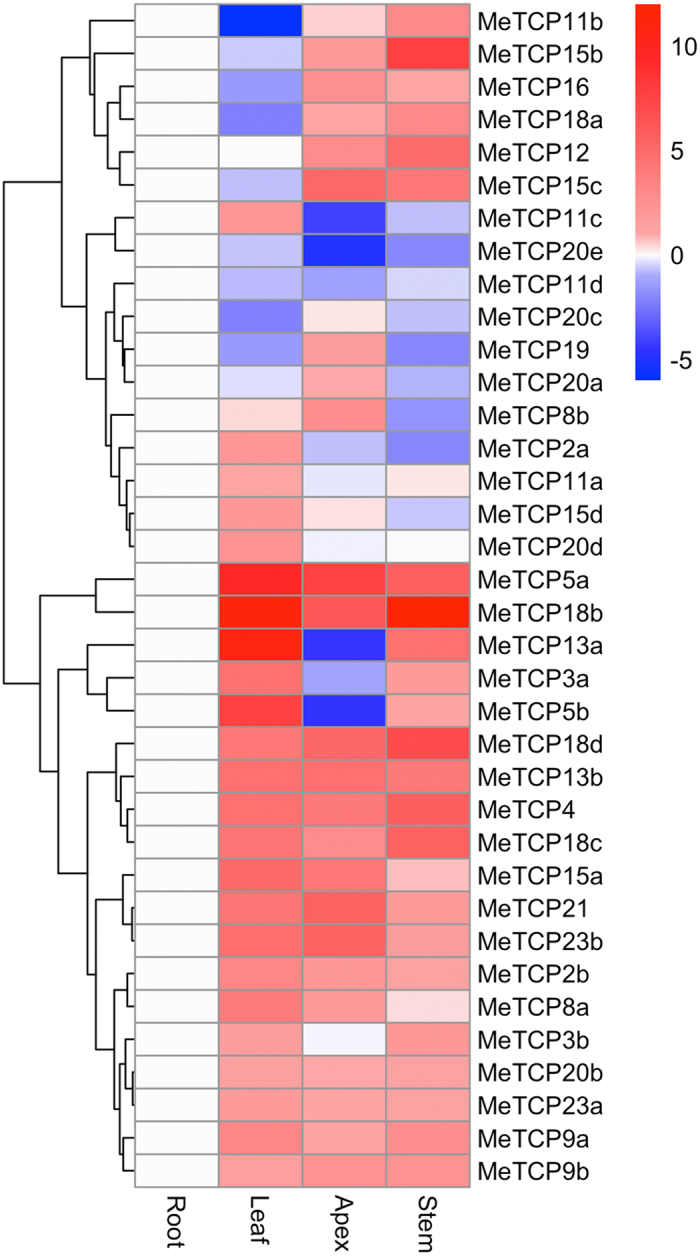
Heatmap representation for expression profiles of 36 *MeTCP* genes across different tissues. The expression levels of *MeTCP* genes were obtain through quantitative real-time PCR. *MeACTIN* was used as the reference gene.

To investigate the potential functions as well as to identify probable functional redundancy through similar expression patterns for the cassava *TCP* genes, the detection of their expression were carried out in different tissues including root, leaf, stem and shoot apex using qRT-PCR. As shown in Fig. 3, it is apparent that the expression levels in different tissues vary widely between the cassava *TCP* genes, as well as between different tissues for individual *TCP* genes, indicating functional specialization among *TCP* gene family members in cassava plant development. Of them, some genes were exclusively highly expressed in a specific tissue. For example, *MeTCP20e* and *MeTCP11d* genes exhibited higher transcriptional abundance in roots as compared to other organs; *MeTCP2a*, *MeTCP3a*, *MeTCP5b*, *MeTCP8a*, *MeTCP13a* and *MeTCP20d* showed specifically high expression in leaves, implying their specific roles in the corresponding tissues. Opposite to the tissue-specific expression pattern of *MeTCP* genes, many genes were more widely and less specifically expressed, such as *MeTCP9b*, *MeTCP13b*, *MeTCP20a*, *MeTCP20b*, and *MeTCP23a* genes, implying that these genes may play regulatory roles at multiple development stages. However, further studies are still needed to unravel the divergent roles of *MeTCP* genes.

To address the conservation and specificity of *TCP* expression pattern beyond species, we compared the expression level of homologous *TCP* gene pairs in these four tissues between *Arabidopsis* and cassava. We found a subset of *TCP* genes were positively correlated with pearson correlation coefficient (PCC) higher than 0.3 between *Arabidopsis* and cassava (Fig. S3–4, such as *MeTCP2a/b* and *AtTCP2*, *MeTCP5a/b* and *AtTCP5*, *MeTCP8a/b* and *AtTCP8*, *MeTCP13a/b* and *AtTCP13*, *MeTCP15b/c* and *AtTCP15*, *MeTCP19* and *AtTCP19 et al*., indicating functional conserved expressional pattern of these genes. In contrast, some *TCP* genes show no correlation or negative correlation between *Arabidopsis* and cassava, such as *MeTCP9a/b* and *AtTCP9*, *MeTCP11a/b/c/d* and *AtTCP11*, suggesting their functions have been diversely changed in different species.

### Expression patterns of *MeTCP* genes in response to hormone treatments

**Figure 4:**
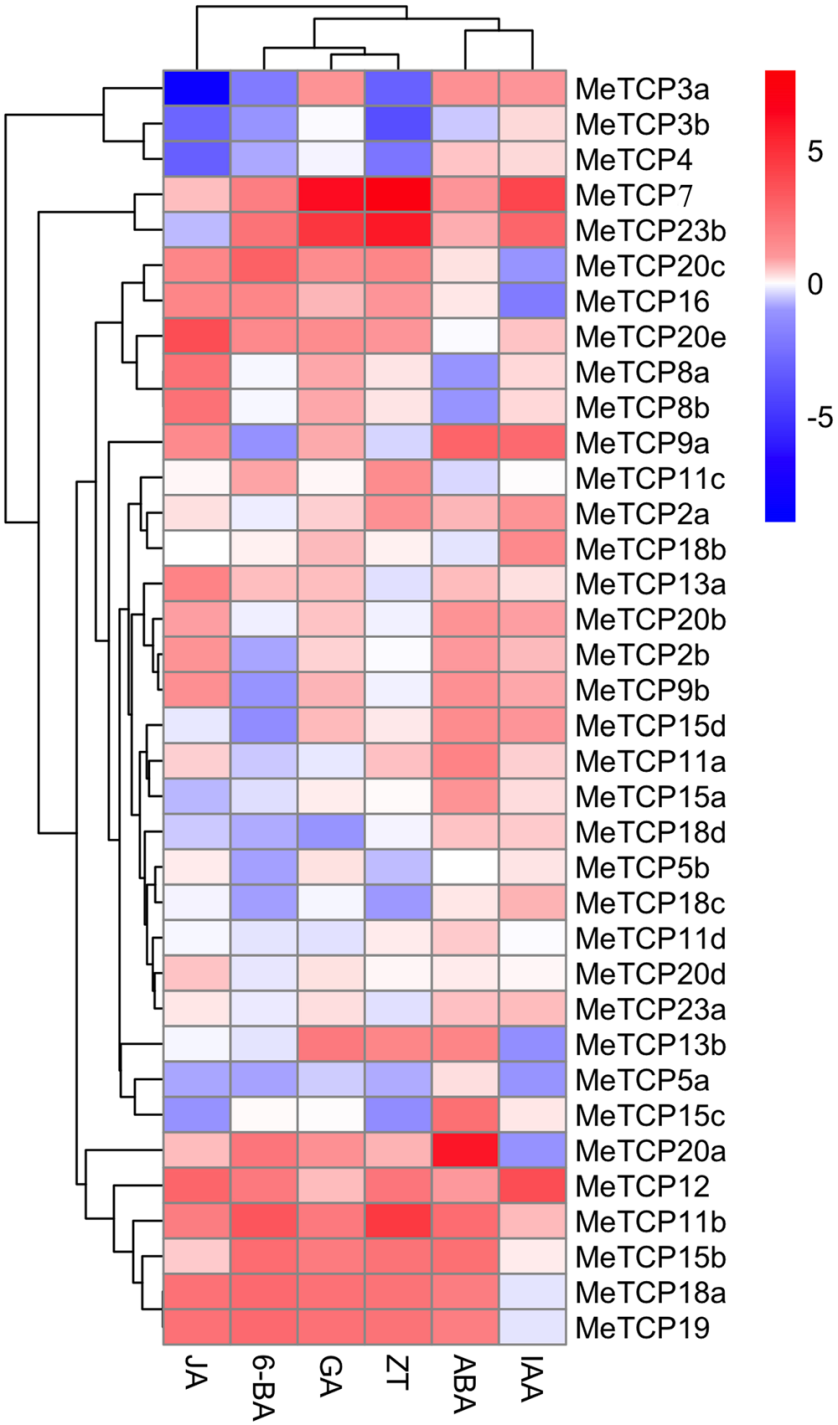
Heatmap representation for expression patterns of *MeTCP* genes under various hormone treatments. The expression profile data of *MeTCP* genes under JA, 6-BA, GA, ZT, ABA and IAA were obtain through quantitative real-time PCR. *MeACTIN* was used as the reference gene.

Multiple studies have been reported that TCP proteins regulate plant development and environmental stress adaption by mediating hormone biogenesis and response^17–19, 21^. To study the total effect of plant hormones on *MeTCP* genes, the expression levels of 36 *MeTCP* genes were detected in response to abscisic acid (ABA), gibberellin (GA3), indole acetic acid (IAA), jasmonate acid (JA), zeatin (ZT) and 6-benzylaminopurine (6-BA) hormone treatments by quantitative RT-PCR (Fig. 4, Supplemental Table S1). In general, hormone treatments resulted in a wide variety of *MeTCP* gene expression profiles. In JA treatment, 15 and 7 *MeTCP* genes were obviously induced and inhibited, respectively. Of them, the most up-regulated gene was *MeTCP20e*, and the most down-regulated gene was *MeTCP3a*. Similarly, 6-BA and ZT treatment led to 12 and 13 *MeTCP* genes were obviously induced, 11 and 5 *MeTCP* genes were inhibited, respectively. In GA treatment, most of *MeTCPs* were significantly induced and only two *MeTCPs* were inhibited. *MeTCP7* and *MeTCP23b* were found to be most up-regulated. As for IAA treatment, 10 and 5 *MeTCP* genes showed dramatic increase and decrease, respectively. *MeTCP7* and *MeTCP16* went through the largest increase and decrease, respectively. ABA plays a crucial role in the adaptive response of plants to abiotic stresses^52^. We found 22 members showed strong sensitivity toward ABA, indicating that these genes may be regulated by ABA signal pathway. Among them, 19 genes had relative high levels of transcript abundance after ABA treatment. Notably, most of *MeTCP* genes responded to at least one treatment; Particularly, *MeTCP11b*, *MeTCP12* and *MeTCP21* responded to all hormone treatments, indicating these genes might play pivotal roles in the cross-talk of hormones, which would be candidates for further research in the field. However, we also found *MeTCP11d*, *MeTCP20a* and *MeTCP23a* were not able to respond to any treatments. Taken together, these results suggest the complicated regulatory mechanism of *MeTCP* genes in response to hormone treatments in cassava.

### Expression profiles of *MeTCP* genes in response to cold and/or drought stress

**Figure 5:**
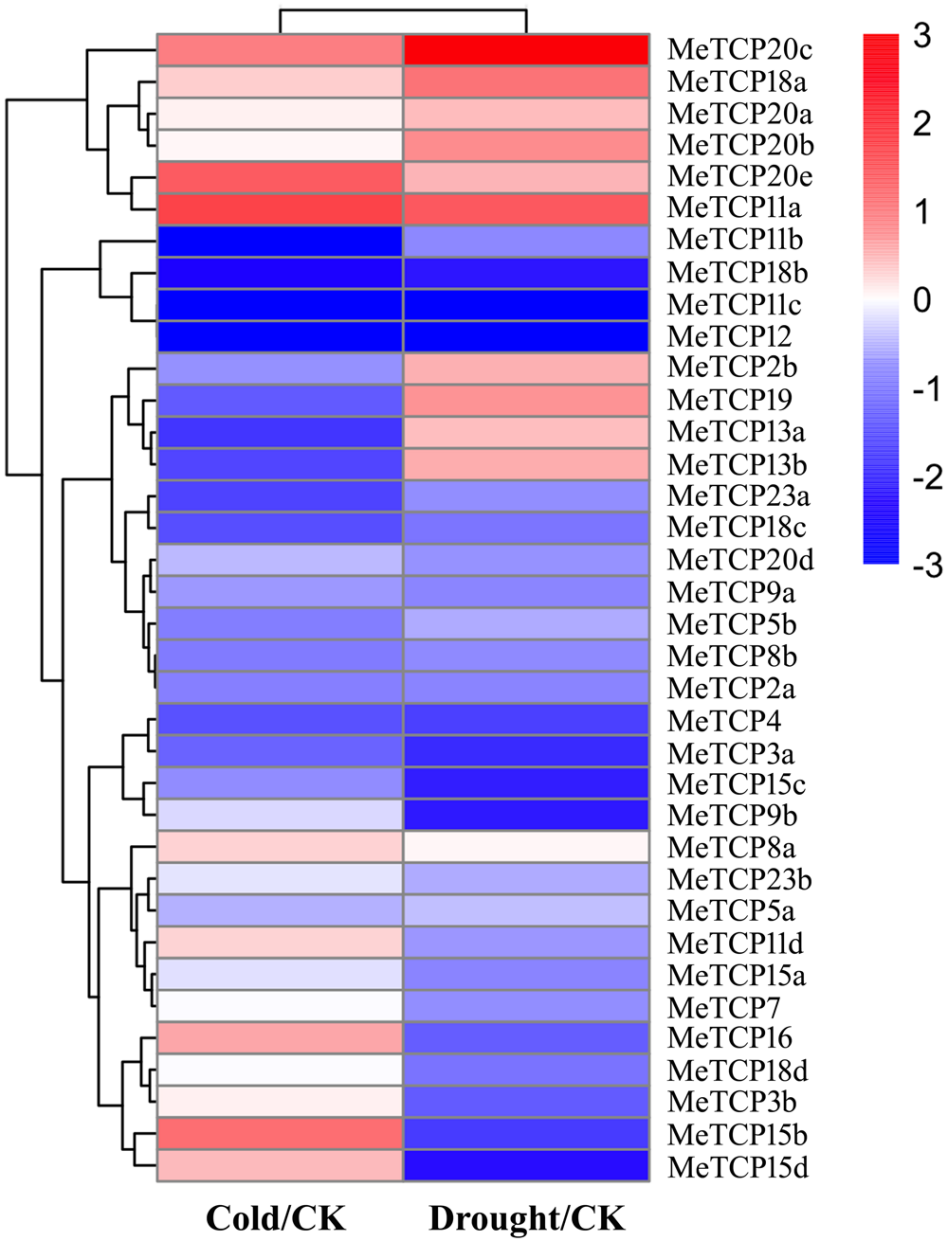
Expression profiles of *MeTCP* genes in leaves and shoot apices after cold and drought treatment. The transcript data generated from three replicates of RNA-seq data. The relative expression values were log2 transformed. The bar represents relative expression values.

Plants are frequently challenged by abiotic stressors such as cold and drought. Recent studies have suggested that TCP proteins are widely involved in signaling and response to environmental stimuli^18, 22^. However, information on the involvement of TCP proteins in stress responses in cassava is limited. To investigate the potential roles of *MeTCP* genes in response to abiotic stresses, cassava seedlings of TMS60444 genotypes were subjected to cold (4 °C) and PEG (20% PEG 6000)-induced drought stress and then the leaves tissues were sampled to extract RNA for subsequent RNA-seq analysis. According to the transcriptome data, 18 (50%) and 24 (66.7%) *MeTCP* genes showed significantly change (fold change >2) under cold and drought treatment, respectively. Among them, 7 (38.9%) and 11 (61.1%) *MeTCP* genes were up- and down-regulated by cold, respectively; 10 (41.7%) and 14 (58.3%) *MeTCP* genes were up- and down-regulated by drought, respectively (Fig. 5, Supplemental Table S2). These results also showed that the number of *MeTCP* genes down-regulated by cold and drought was greater than that were up-regulated, suggesting the comprehensive response of *MeTCP* genes to cold and/or drought at transcriptional levels. There were 23 *MeTCP* genes in total differential expressed under both cold and drought treatments, which were categorized into 4 different classes: concordant response to cold and drought, discordant response, cold-specific and drought-specific. Among concordant response, *MeTCP20c/20e/11a* and *MeTCP18b/11c/12* were co-induced and co-repressed by two kinds of stresses, respectively. However, in discordant response class, three genes (*MeTCP15d*, *MeTCP15b* and *MeTCP16*) showed increased expression pattern under cold treatment, whereas down-regulated after drought treatment. By contrast, the expression levels of four genes (*MeTCP2b*, *MeTCP19*, *MeTCP13a* and *MeTCP13b*) were induced by drought treatment, but were repressed or unaltered after cold treatment. Meanwhile, *MeTCP8a/5a* and *MeTCP20a/20b/9b/18d/3b* were specifically response to cold and drought stress, respectively. Generally, the expression levels of *MeTCP* genes in response to cold and drought stresses were dramatically changed, implying their putative roles in stress tolerance.

### Expression analysis of *MeTCP* genes under various abiotic stress treatments

**Figure 6:**
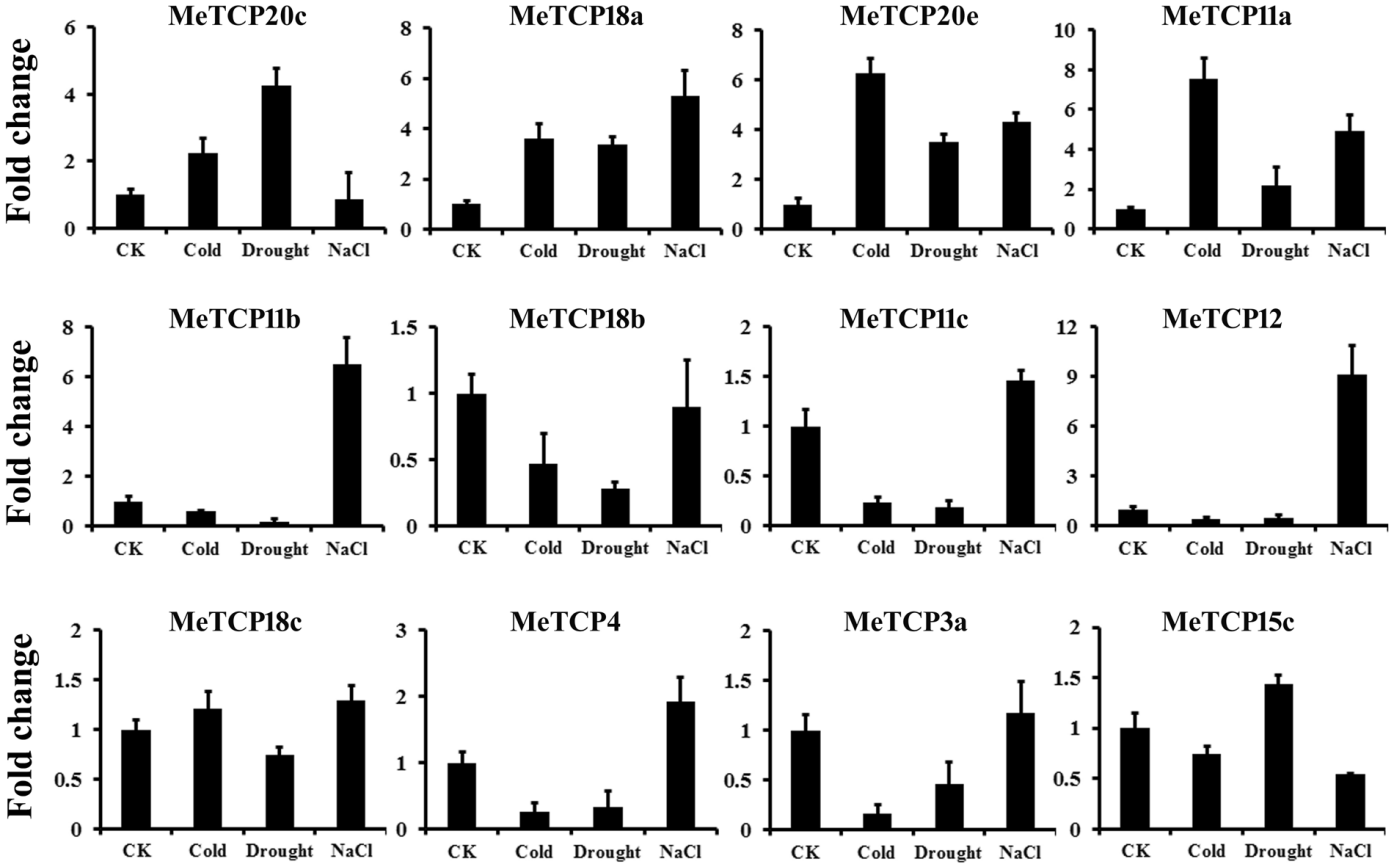
Confirmation of the expression patterns of cold- and drought-responsive *MeTCPs* using qRT-PCR. The expression patterns of *MeTCP* under cold, drought and salt stress. The values shown are the means ± standard deviation of three replicates. *MeACTIN* was used as the reference gene.

To further assess the response of *MeTCP* genes to various abiotic stresses and related signaling pathway at transcriptional levels, 12 *MeTCP* genes (*MeTCP20c*, *MeTCP18a*, *MeTCP20e*, *MeTCP11a*, *MeTCP11b*, *MeTCP18b*, *MeTCP11c*, *MeTCP12*, *MeTCP18c*, *MeTCP4*, *MeTCP3a* and *MeTCP15c*) induced or repressed by cold and drought stresses based on RNA-seq data were chosen for further examination of their expression patterns after cold, drought and salt treatments (Fig. 6). We found most of the analyzed genes exhibited differential expression in response to at least one stress treatment, implying their putative roles in these stresses tolerance. Overall, in the cold, drought and salt conditions, expression of *MeTCP20c*, *MeTCP18a*, *MeTCP20e* and *MeTCP11a* genes were significantly up-regulated, and the largest expressional change of these genes were usually observed when responding to cold and/or drought treatments. This observation is well consistent with the RNA-seq data. 6 genes (*MeTCP11b*, *MeTCP18b*, *MeTCP11c*, *MeTCP12*, *MeTCP4* and *MeTCP3a*) were down-regulated following cold and drought treatments, among which, *MeTCP12* underwent the greatest change of mRNA expression levels. Interestingly, the expression levels of all these *MeTCP* genes showed obviously increase under salt treatment, suggesting they may play different roles in response to the three stresses. In addition, not too many changes were observed in *MeTCP18c* and *MeTCP15c* genes when any of three stresses were carried out. These data show the potential of some *MeTCP* genes for enhancing adversity resistant capacity in cassava.

### Analysis of the regulatory cis-elements in the promoter of cold- and drought-responsive *MeTCPs*

**Figure 7:**
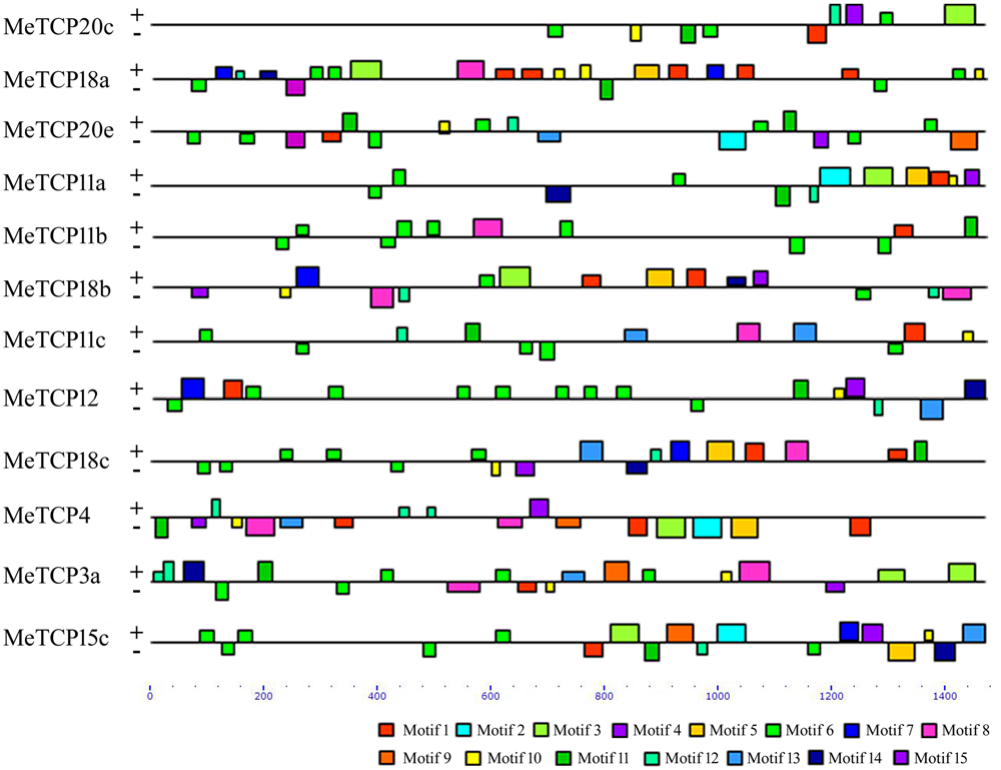
The conserved DNA sequence motifs analysis of cold- and drought-responsive *MeTCP* promoters. The conserved motifs in the TCP promoters were identified using MEME program. Each motif is indicated with a specific color.

The altered expression of 12 *MeTCPs* that were co-induced/repressed by cold and drought stresses indicates that they may be regulated by key stress regulatory genes. In order to elucidate the mechanism of transcriptional regulation of these genes, analysis of their promoter region was performed for the cis-elements. A 1.5 kb sequence upstream to the open reading frame of *MeTCP20c*, *MeTCP18a*, *MeTCP20e*, *MeTCP11a*, *MeTCP11b*, *MeTCP18b*, *MeTCP11c*, *MeTCP12*, *MeTCP18c*, *MeTCP4*, *MeTCP3a* and *MeTCP15c* was identified and subjected to MEME analysis. A number of common cis-acting elements were identified in the proximal promoters (Fig. 7, Fig. S5). All the identified promoters had motif 1, and most of them also contained motif 3 and 10, except for *MeTCP20e*, *MeTCP11b*, *MeTCP11c*, *MeTCP12* and *MeTCP18c*, suggesting that these commonly present cis-acting elements may be involved in stress response. Notably, motifs such as 9, 13, 14 and 15 are more present in the promoters of *MeTCP* which are down-regulated by both cold and drought stresses, suggesting that these motifs may play key roles in the regulation of *MeTCP* members.

## Discussion

Adverse environmental conditions, such as cold and drought stress, impose severe effects on the plant growth, development and limit crop productivity and yield^53^. TCP transcription factors are a class of plant-specific transcription that play very important roles during plant development and abiotic stress responses^22^. To our knowledge, although a range of TCP family members have been described in various species^35, 36, 40–45, 54^, no studies have been performed on *TCP* genes in cassava. Additionally, the mechanisms of cassava responds to abiotic stress are poorly understood. This background knowledge prompted us to identify the full complement and expression profile of this important gene family during development and under abiotic stresses in cassava.

In the present study, a comprehensive set of 36 non-redundant *TCP*-encoding genes were identified and characterized from the current version of the cassava genome. Previous studies have identified 24 TCP genes in *Arabidopsis* ^33^, 22 in rice^34^, 38 in cotton^35^, 30 in tomato^36^, and 27 in watermelon^43^. The amplification of TCP gene family members in cassava can be explained by its larger genome size (~760 Mb) compared to that of *Arabidopsis* (~125 Mb) and gene duplication events in this family. Evolutionary analysis indicated that the cassava *TCPs* could be clustered into 8 subgroups, which is minimal different with previous evolutionary classification of TCPs in cotton and watermelon^35, 43^ (Fig. 1). The phylogenetic tree showed obvious differences in number of *MeTCPs* and ratio of *MeTCPs*/*AtTCPs* among subgroups. The change in the ratio of *MeTCPs*/*AtTCPs* suggested that the *MeTCP* family had undergone lineage-specific expansion and functional divergence during the course of evolution. Phylogenetic tree also showed that group C contained three cassava members and two *Arabidopsis* members, but there were no *TCP* from rice. On contrast, *TCPs* in subgroup A expanded in monocots but not in *Arabidopsis*, indicating that *TCP* genes of these subgroups expanded in a species-specific manner from common ancestral genes that were present prior to the diversification of the monocot and dicot lineages. The classification of TCP protein was further supported by gene structure analysis and conserved protein motif analysis. Gene structure analysis showed that the majority of *MeTCP* genes within the same subgroup exhibited very similar gene structure in terms of exon length and intron number. Furthermore, conserved protein motif analysis indicated that all the MeTCP proteins contained typical TCP domain, except MeTCP20c, which contains a truncated TCP domain. Similar to the gene structure, most subgroups of MeTCPs also exhibited conserved motif composition, with several motifs observed in some MeTCP subgroups, such as motif 4 and 17 for subgroup D, motif 7 for subgroup A (Fig. 2c). These unique motifs may contribute to the specific function of these subgroup members. In general, the majority of *MeTCP* genes in the same subfamilies are evolutionarily conserved, which supports their close evolutionary relationship and the classification of subgroups.

The expression pattern analysis of *MeTCP* genes helps us to assess their possible functions and provide a solid foundation for future functional studies. Generally, similar to the previous study, *MeTCP* genes exhibited greatly differential expression patterns across a variety of tissues, not only among subgroups but members within the same subgroups (*MeTCP5a* and *MeTCP5b*, *MeTCP13a* and *MeTCP13b*), suggesting that these *MeTCP* genes may function diversely in various tissues (Fig. 3). On the contrary, some *MeTCP* genes with extremely high sequence identity (*MeTCP2a* and *MeTCP2b*, M*eTCP18c* and *MeTCP18d*) showed conserved expression patterns (Fig. 3), implying they may play a redundant role in regulating plant growth.

To date, the role of plant hormones in regulating plant growth, development, and abiotic stress responses by modulating gene expression is well established^52, 55^. To our knowledge, although the relationship between TCP proteins and hormones has been widely known, such as cytokinins, JA and GA, the dynamic and spatially expression patterns of *MeTCP* genes response to various hormones was still obscure. Our current results revealed that the majority of *MeTCP* genes detected here displayed distinct changes under different hormone treatments. To expect, most of *MeTCP* were up-regulated or down-regulated by cytokinins (ZT and 6-BA). *MeTCP15b* and *MeTCP23b*, orthologs of *AtTCP15* and *AtTCP23*, respectively, had high expression levels under ZT and 6-BA treatments. In *Arabidopsis*, cytokinin treatments induce *TCP15* transcription and promote *TCP15* (and *TCP14*) protein activation by post-translational modification, which in turn promote cytokinin responses^56^. *MeTCP4*, *MeTCP20b*, and *MeTCP20e*, orthologs of *AtTCP4* and *AtTCP20*, respectively, had altered expression patterns under JA. In young leaves of *Arabidopsis*, *AtTCP20* repressed the transcription of *LIPOXYGENASE2* (*AtLOX2*) gene, which is involved in JA synthesis and promotes leaf senescence, while this negative control is antagonized by *AtTCP4* as the leaf matures^19^. It is noteworthy that 22 *MeTCPs* were response to ABA, indicating these genes might function as key mediators of stress responses through ABA signaling pathways. Taken together, these results suggested that MeTCPs play potential regulatory roles by modulating phytohormone signaling in plant development or in the responses to stresses. Therefore, it will be particular important to further investigate the potential function of *MeTCP* genes in hormone signaling in the future.

Previous reports have shown that plant *TCP* genes are involved in plant growth and development, as well as abiotic stress responses under normal and stressed growth conditions^22^. In cassava, our data showed that over half of the *MeTCP* genes were significantly upregulated under cold and drought condition. Among them, 4 genes were up-regulated, and 8 genes were down-regulated by both cold and drought stress. Moreover, combined analysis of expression correlation and promoter content has revealed that most of these *MeTCP* genes exhibited differential expression in response to more than one stress treatments, suggesting the wide involvement of *MeTCP* genes in environmental adaptation. Previous reports have shown that knockdown of miR319-dependent *TCPs* (by constitutive miR319 overexpression) increases drought and salinity stress tolerance in bentgrass^20^. Our data showed that *MeTCP3a* and *MeTCP4*, targets of miRNA319, had altered expression patterns under cold, drought and salt stress, suggesting that these genes might play important roles under abiotic stress conditions in cassava. These data indicated that MeTCP might function in resistance to abiotic stresses in cassava.

In conclusion, we identified 36 *TCP* transcription factor genes from cassava. Phylogenetic analysis of cassava, *Arabidopsis*, and rice indicated that these *MeTCP* genes could be divided into 8 groups, which is supported by further conserved protein motif, and gene structure analyses. Although nearly all the *MeTCP* genes were expressed in the examined tissues, some genes were up-regulated in one or several specific organs. mRNA accumulation was altered by a variety of hormone treatments (ABA, IAA, GA, JA, ZT and 6-BA), environmental conditions (drought, high salinity, and low temperature). These results suggested that MeTCP family proteins play critical roles in maintaining cassava normal growth under normal or stress conditions through complicated mechanisms. Thus, additional studies on the detailed functions of each gene are warranted in cassava.

## Materials and Methods

### Identification and bioinformatics analysis of candidate genes

To identify potential members of the cassava TCP protein family, The *Arabidopsis* TCP protein sequences were used as seed queries in BLASTp searches against the cassava database (Phytozome: http://www.phytozome.net/cassava.php)^57^. The TCP domain (PF03634, Pfam; http://pfam.sanger.ac.uk/)^46^ was also employed as query to perform a blast search against the same genome database. The identified MeTCP proteins were renamed as MeTCP2 to MeTCP23 according to the *Arabidopsis* TCP proteins with highest sequence similarity. Information on *MeTCP* genes, including exons and introns number, open reading frame (ORF) and amino acid (AA) lengths, was obtained from Phytozome database. The molecular weight, theoretical isoelectric point (PI) and grand average of hydropathy (GRAVY) of the MeTCP proteins were investigated using ExPASy online tools (http://web.expasy.org/protparam/).

### Analysis of phylogenetic relationships and gene structure

Multiple sequence alignments were applied to confirm the conserved domains of predicted MeTCP proteins. The Clustal × 2.0^58^ was employed to align the full-length MeTCP proteins from cassava, *Arabidopsis* and rice. Then, the bootstrap neighbor-joining evolutionary tree was created by MEGA 6.0 software^59^ with 1000 bootstrap replicates based on the sequence alignments. The exon-intron organization of *MeTCP* genes was determined by comparing the coding DNA sequence (CDS) with its corresponding genomic sequences using the Gene Structure Display Server (GSDS) software (http://gsds.cbi.pku.edu.cn/)^60^.

### Identification of conserved motif of MeTCP proteins and promoters

By using the Multiple Expectation maximization for Motif Elicitation (MEME) program (http://meme.nbcr.net/meme/cgi-bin/meme.cgi), the conserved motifs in full-length cassava MeTCP protein sequences were identified with the following parameters: maximum number of motifs was 20 and the optimum width of motifs was set between 10 and 50^49^. The identified protein motifs were further annotated with ScanProsite^50^. *MeTCP* promoter sequences in cassava were submitted to online MEME program for identification of conserved motifs. The optimized MEME parameters were as follows: any number of repetitions and maximum number of motifs-15.

### Plant materials and hormone/stress treatment

Cassava (*Manihot esculenta*) cultivar (TMS60444) was used in the present study. Segments cut from cassava stems were inserted into MS plates in a greenhouse at 26 ± 2 °C, with a photoperiod of 16 h light and 8 h dark. All hormone and environmental treatments were conducted when uniform-sized seedlings developed two fully opened trifoliate leaves (approximately two weeks after sowing). For hormone treatment, 14-day-old cassava seedlings were soaked in liquid MS medium with 100 μM indole acetic acid(IAA), 100 μM gibberellin (GA3), 100 μM Methyl jasmonate (MeJA), 100 μM abscisic acid (ABA),100 μM zeatin (ZT) and 100 μM, 6-benzylaminopurine (6-BA) for 3 h, respectively, and then the young leaves and shoot apex from at least ten separate seedlings/plants were harvested. Seedlings soaked in liquid MS medium without any hormone were used as control. For cold treatment, seedlings were placed at 4 °C for 24 h, and then the young leaves and shoot apex were collected for RNA isolation. For drought and salt stress treatment, cassava seedlings were treated with 20% PEG6000 and 100 mM NaCl, and harvested at 6 h after treatment, respectively. In all cases, parallel and untreated plants at the same stage were used as controls. All samples harvested were flash-frozen in liquid nitrogen, and stored at −80 °C until RNA isolation.

### RNA isolation and expression analysis

Total RNA was extracted from 0.1 g of tissue by using Plant RNA kit (OMEGA), following the manufacturer’s instructions. Reverse transcription reactions were performed using 5 μg of RNA with PrimeScript RT reagent kit with gDNA Eraser (TIANGEN, Beijing, China). Quantitative reverse transcription PCR (qRT-PCR) was performed as described elsewhere^61^, the PCR conditions were as follows: pre-incubation at 94 °C for 5 min, followed by 40 cycles at 94 °C for 10 s, 60 °C for 10 s, 72 °C for 30 s. After amplication was complete, a melting curve was obtained by holding at 95 °C for 5 s and then at 65 °C for 15 s, followed by heating slowly at 0.1 °C/s to 95 °C. Real-time PCR was performed with a Bio-Rad real-time thermal cycling system using SYBR® Premix Ex Taq™ II (TaKaRa, Japan) to assess gene expression levels. The relative expression levels of each gene were calculated by the 2^-ΔΔCt^ method. The cassava actin gene was used as internal control for normalization. The primers used are listed in Supplemental Table S3. *MeTCP* genes that were up- or down-regulated by at least two-fold were considered as differentially expressed. The experiments were performed in triplicate.

### Expression correlation analysis of level between cassava and *Arabidopsis*

The expression levels of *AtTCPs* in different tissues were extracted from the microarray data^62^. To compare the microarray gcRMA value of AtTCPs with qPCR relative value of MeTCPs, all values were normalized to z-score, and pearson correlation coefficient were performed for each homologous gene pairs.

### Transcriptome analysis

Cassava shoot apices and youngest leaves of TMS60444 under normal conditions, cold and drought treatments were used to isolated total RNA for transcriptome analysis. As previously described^61^, the total RNA isolation, whole transcriptome libraries preparation and deep sequencing were performed by the Annoroad Gene Technology Corporation (Beijing, PR China). A total of 140 gigabase in-depth sequencing of library was performed initially on a HiSeq. 2500 instrument that generated paired-end reads with 125 nucleotides. Data analysis was carried out by previously described^61^. The generated transcriptomic data has been submitted to Sequence Read Archive (SRA) in NCBI with the accession number SRP101302.

## Electronic supplementary material

Supplementary Figures

Supplementary Table
