# Supplementary material for: Phylogeny and expression pattern analysis of TCP transcription factors in cassava seedlings exposed to cold and/or drought stress
Source: Sci Rep. 2017 Aug 30;7:10016. doi: 10.1038/s41598-017-09398-5 (PMC5577251; doi:10.1038/s41598-017-09398-5)

Supplementary information

Phylogeny and expression pattern analysis of TCP transcription factors in cassava seedlings exposed to cold and/or drought stress

Ning Lei[2†], Xiang Yu[3,4†], Shuxia Li[1†], Changying Zeng[1], Liangping Zou[1], Wenbin Liao[1] & Ming Peng[1*]

[1]Institute of Tropical Bioscience and Biotechnology, Chinese Academy of Tropical Agricultural Sciences, Haikou 571101, China; and

[2]Institute of Tropical Agriculture and Forestry, Hainan University, Haikou, 570228, China; and

[3]National Key Laboratory of Plant Molecular Genetics and National Center for Plant Gene Research (Shanghai), Institute of Plant Physiology and Ecology, Shanghai Institutes for Biological Sciences, Chinese Academy of Sciences, Shanghai 200032, China; and

[4]Current address: Department of Biology, University of Pennsylvania, Philadelphia, PA 19104, USA

[†]These authors contributed equally to this work.

[*]Corresponding author: Ming Peng, pengming@itbb.org.cn

Figure S1. Phylogenetic relationships of TCP transcription factors built with the minimal evolution method. A total of 36 *MeTCPs* from cassava, 24 *AtTCPs* from Arabidopsis and 22 *OsTCPs* from rice were used to construct the Minimum-Evolution tree by MEGA 6.0 with 1000 bootstrap based on the full length sequences of *TCPs*.

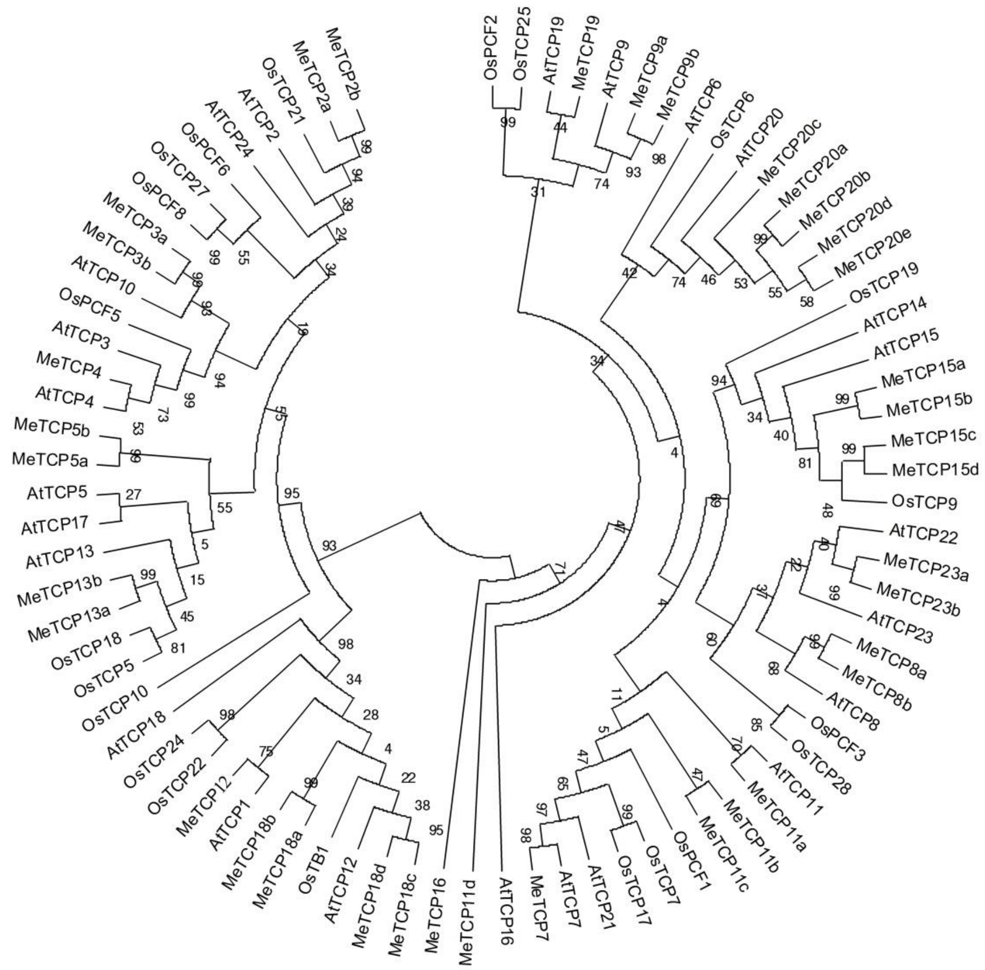

Figure S2. Protein motifs of MeTCPs identified by MEME.

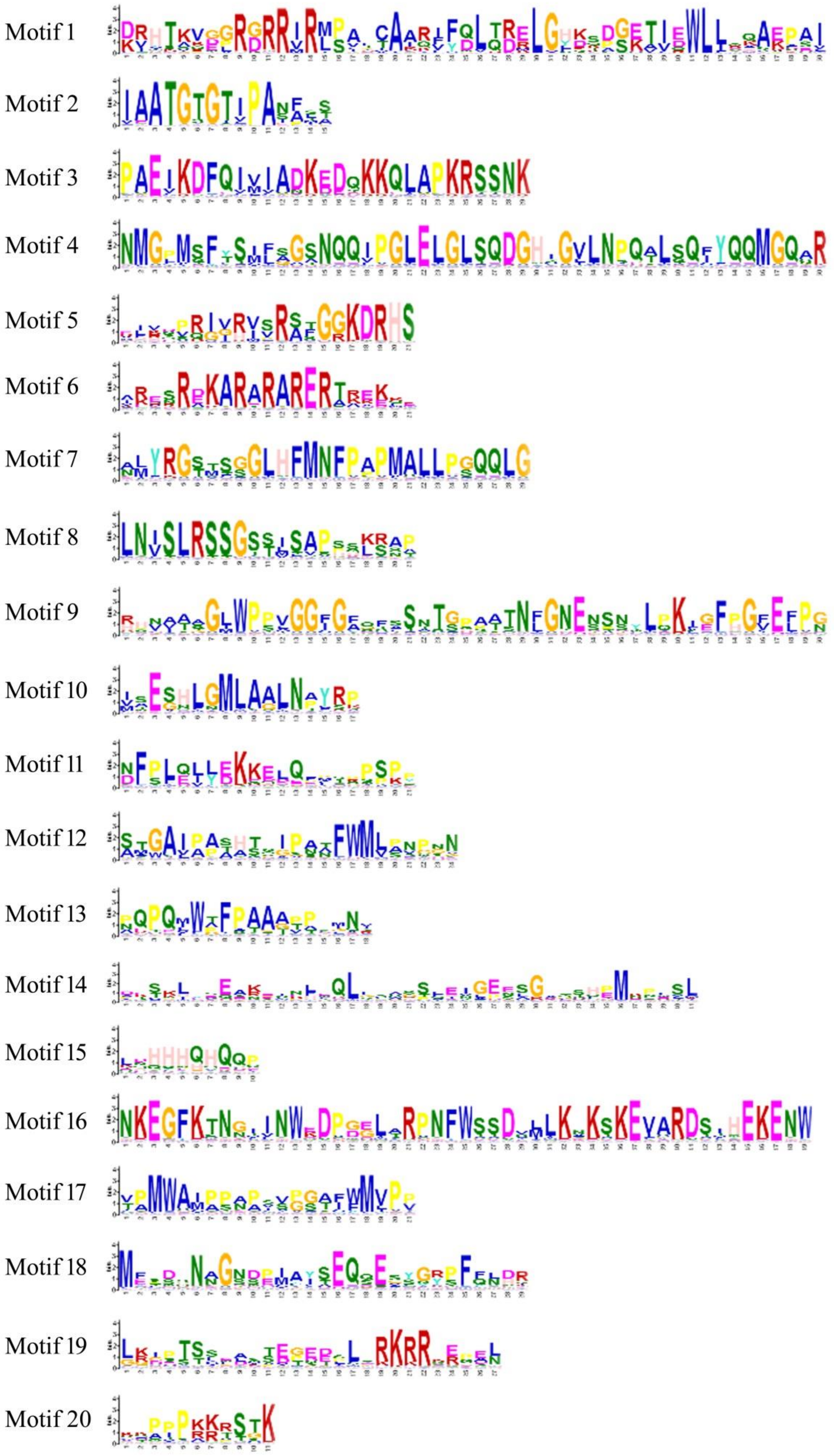

Figure S3. Scatterplot showing the correlation of TCP expressional value (z-score) between Arabidopsis and Cassava homologous genes (Part 1). **R indicates root,L indicates leaf, A indicates apex, and S indicates stem, respectively. r indicates pearson correlation coefficient.**

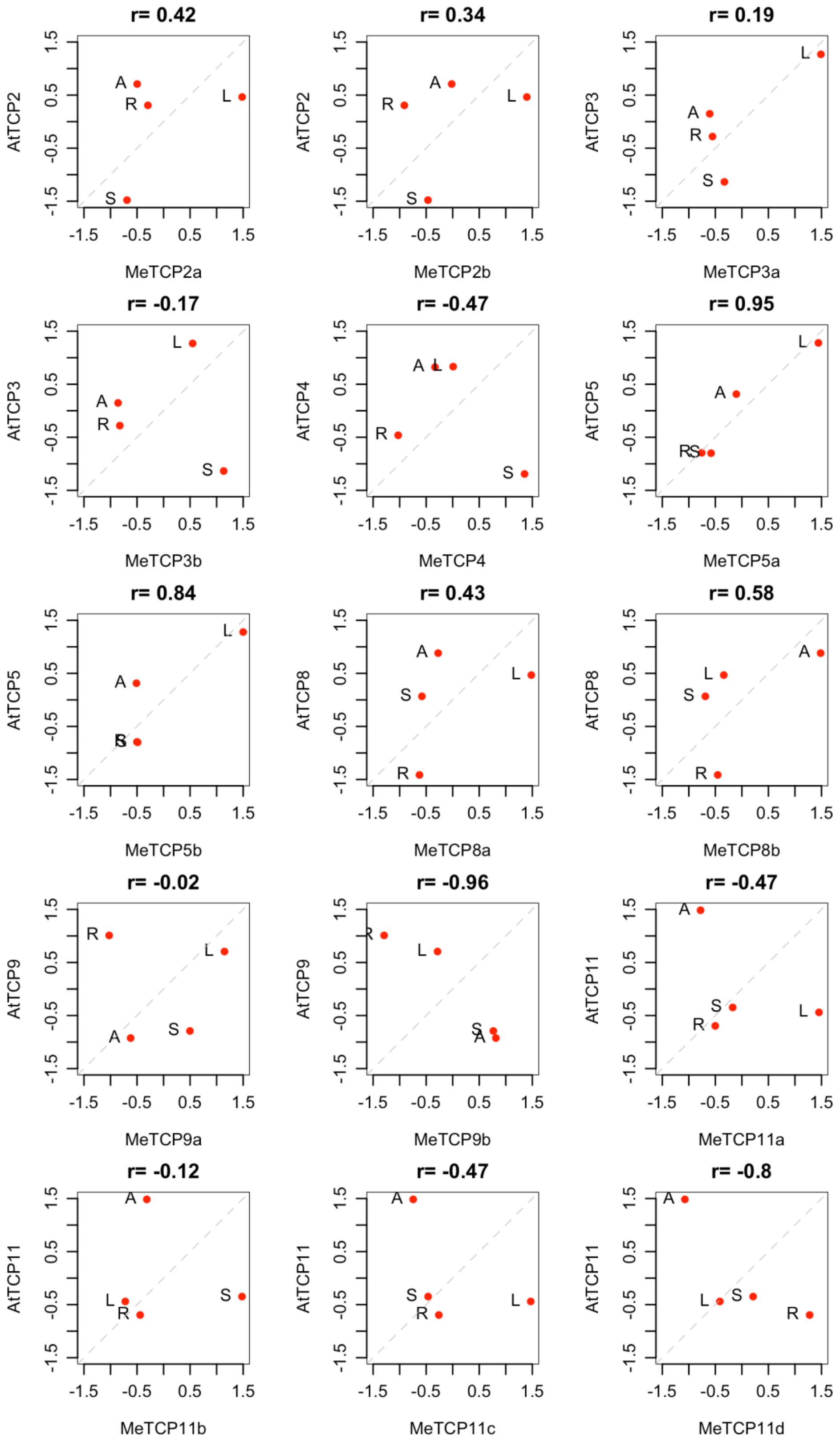

Figure S4. Scatterplot showing the correlation of TCP expressional value (z-score) between Arabidopsis and Cassava homologous genes (Part 2). **R indicates root,L indicates leaf, A indicates apex, and S indicates stem, respectively. r indicates pearson correlation coefficient.**

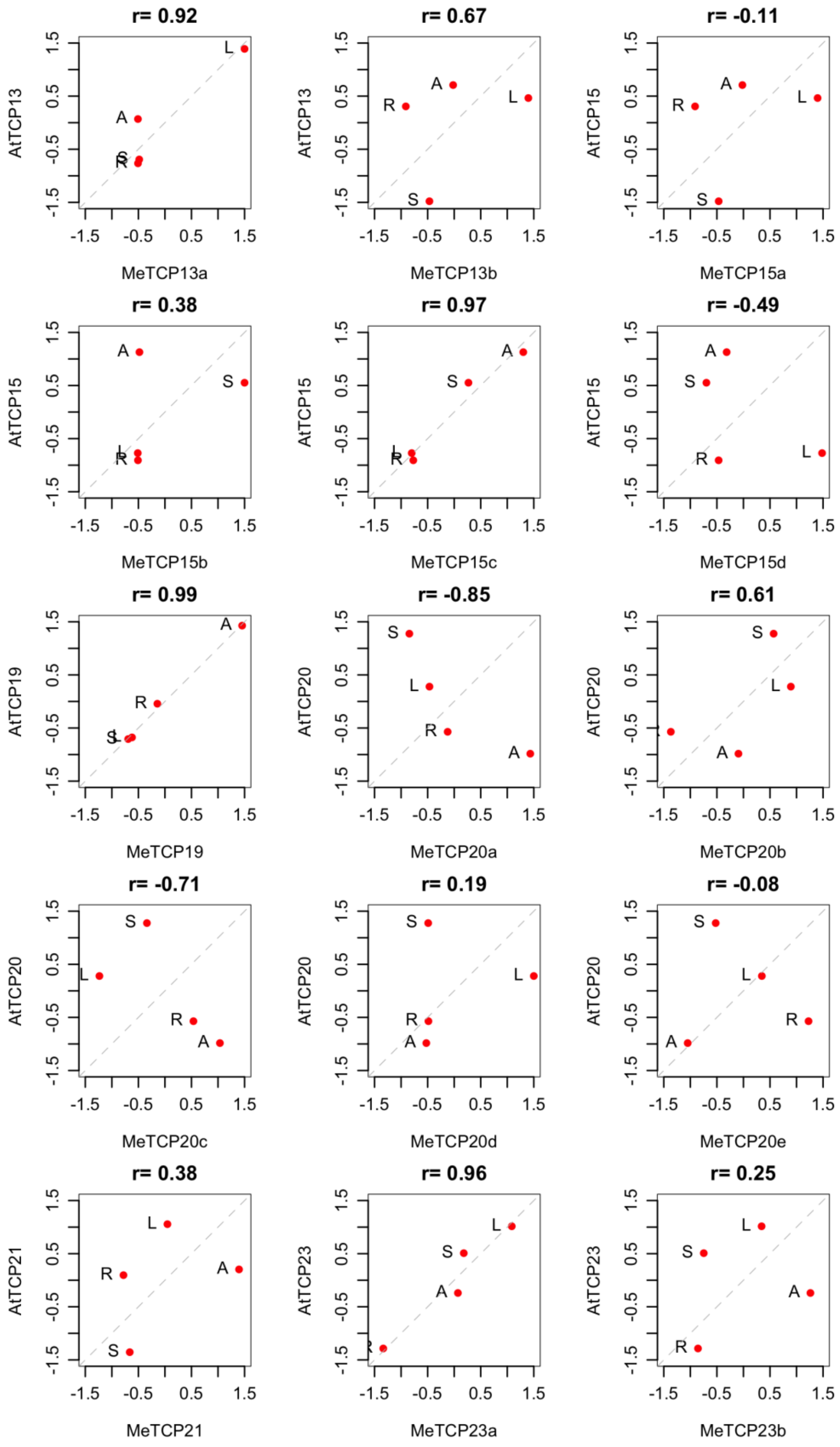

Figure S5. Motifs identified in the promoter of *MeTCPs* by MEME.

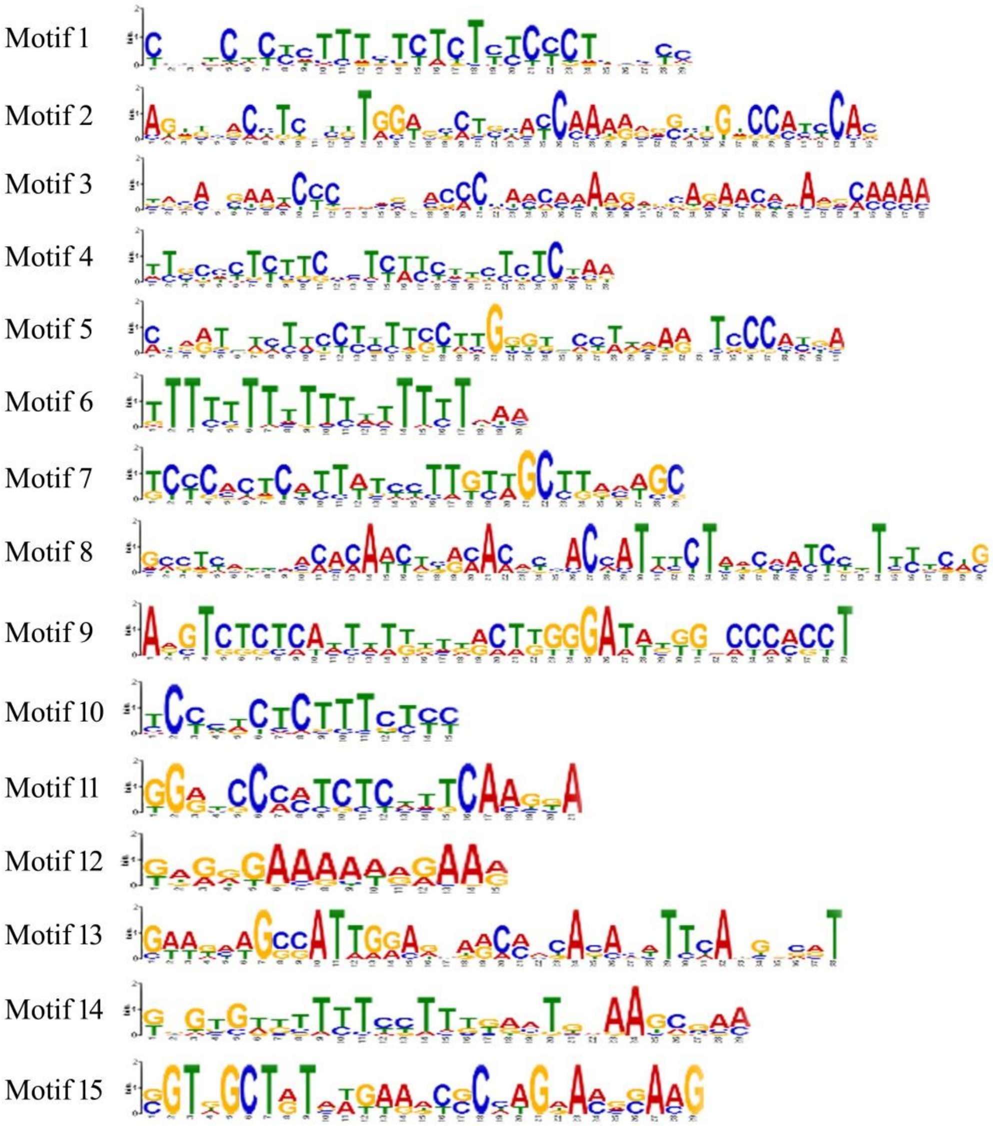

Supplement: Supplementary file 1 — Supplementary Figures [file 41598_2017_9398_MOESM1_ESM.pdf]
